# Alumni Medical Association of Chicago Medical College

**Published:** 1883-07

**Authors:** 


### ARTICLE XIV.

### ALUMNI MEDICAL ASSOCIATION OF CHICAGO MEDICAL COLLEGE.

THE Alumni Medical Association of the Chicago Medical College held their annual meeting at 10 o'clock A. M. of March 27, 1883, Dr. M. P. Hatfield, the President in the chair. The necrologist reported upon the death of the following members:

Craft Green, class of 1882; Geo. H. Wright, 1879; Green B.

Hoblit, 1869; Chas. Ashworth, 1869; Gordon M. Conville, 1868; E. C. Dickenson, 1860; J. D. Hogan, 1876; J. L. Quirk, 1867; Clarence J. Rivenberg, 1878; Isaiah W. Grist, 1871; C. T. Listenberg, 1873; Wm. T. Howard, 1876; Wm. E. Gallop, 1880.

H. M. Scudder, M.D., D.D., delivered a very instructive address on "The State of Medical Science in India."

The following prizes were presented by Rev. D. Arthur Edwards, of the *Northwestern Christian Advocate*, for the Association:

Alumni Prize of fifty dollars, for the highest average scholarship during three years' attendance at the college—taken by N. S. Davis, Jr.

Edward's Prize, set of Wood's library, for best scholarship during prior and middle years—taken by A. C. Helm.

Lena Prize, two volumes on surgical subjects, for best anatomical preparation—taken by Stanley P. Black, class of 1883.

President's Prize, two volumes on "Diseases of Children"—divided between Wm. H. Graves and Jas. Miller.

Faculty Prizes, for best and second best graudating theses:

1st. Lusk's "Science and art of Midwifery," to N. S. Davis, Jr.

2nd. "Hyde on Diseases of the Skin," to W. E. Burbank.

Honorable mention: G. W. Post, O. L. Schmidt, G. A. Wells, E. G. Epler, Jas. E. Henderson.

Shafer Prizes:

1st. For best scholarship in anatomy in junior class, gold medal—to Theo. H. Swain.

2nd. For best scholarship in anatomy, in the middle class, a volume of medicine—to Wm. Elliotte.

3rd. For best competitive examination in anatomy, "Quain's Dictionary of Medicine," and the Prosectorship—to H. R. Frothingham.

Dudley Prizes—six cases of instruments—for the six best reports of lectures on gynæcology—to J. E. Henderson, Geo. W. Post, J. F. McAuley, Frank P. Peck, E. G. Eppler and Chas. Davison.

The following list of officers was elected for the ensuing year :

President, D. A. Sheffield, of Apple River.

1st Vice-Pres., A. H. Burr, Chicago.

2nd Vice-Pres., N. S. Davis, Jr., Chicago.

Necrologist, F. E. Waxham, Chicago.

Secretary and Treasurer, H. T. Byford, Chicago.

The following committees were appointed :

On Library—M. P. Hatfield, E. Wyllys Andrews, and the acting Secretary.

To coöperate with the officers of the association in making preparations for the quarter-centennial celebration of the foundation of the college, next year—M. P. Hatfield, E. J. Doering, and F. Billings.

To report on the suggestions contained in the President's address, and upon a life-membership fee—Roswell Park, E. J. Doering, and D. A. K. Steele.

---

### Reviews and Book Notices.

---

ARTICLE XV.—PAY HOSPITALS AND PAYING WARDS THROUGHOUT THE WORLD. BY HENRY C. BURDETT, Condon. Presley Blakiston, Philadelphia, 1880.

The author is evidently familiar with hospital management, and in his little volume has given a succinct account of the origin and growth of the hospital system ; has enlightened us as to the pay hospitals of France, Switzerland, Germany, Austria, Spain, Italy, Sweden, Norway, Canada, Ireland, England, and finally our own country. He delineates well, and satisfactorily accounts for the abuses of hospitals, and claims that the only proper kind of a hospital is a pay hospital. He also very properly sets forth the advantages to be derived by both the laity and medical profession in the establishing of convalescent hospitals.

